# Delayed presentation of food protein-induced enterocolitis syndrome (FPIES) to okra in a toddler

**DOI:** 10.1186/s13223-024-00871-1

**Published:** 2024-02-03

**Authors:** Hunter Hall, Sara Anvari, Fallon Schultz, Olubukola Ojuola, Nicholas L. Rider

**Affiliations:** 1 Department of Biology, Liberty University, Lynchburg, VA USA; 2 Division of Immunology, Allergy and Retrovirology, Baylor College of Medicine, Texas Children’s Hospital, Houston, TX USA; 3 International FPIES Association, Point Pleasant Beach, NJ USA; 4 Division of Pediatrics, Liberty University College of Osteopathic Medicine, Collaborative Health Partners, Lynchburg, VA USA; 5 Department of Health System & Implementation Science, Virginia Tech Carilion School of Medicine, 1 Riverside Circle, 249, Roanoke, Virginia 24016 USA

**Keywords:** Food protein-induced enterocolitis syndrome (FPIES), Non-immunoglobulin E (IgE), Vomiting, Okra, Food allergy

## Abstract

**Background:**

Food protein-induced enterocolitis syndrome (FPIES) is a non-immunoglobulin E (IgE) -mediated food allergy predominantly observed in infants and characterized by the delayed onset of vomiting following ingestion of a trigger food. An increase in research and clinical consideration of FPIES has led to the discovery of unique deviations from the standard FPIES triggers and presentations.

**Case presentation:**

A 34-month-old female patient with a history of consuming okra daily presented to medical attention after developing classic FPIES symptoms to okra beginning at 14-months of age.

**Conclusions:**

Recently, awareness about the varied nature of FPIES clinical presentation has come to light. This case is the first to describe FPIES to the fruit okra that developed over a 12-month time span after previously tolerating the food. This case serves to emphasize the importance of understanding the range of FPIES symptoms to improve recognition and expedite best practice recommendations.

## Background

Food protein-induced enterocolitis syndrome (FPIES) is a non-immunoglobulin E (IgE) -mediated food allergy predominantly observed in infants and characterized by the delayed onset of emesis (1–4 h) following ingestion of a trigger food [1, 2]. Other accompanying symptoms may include pallor, lethargy, diarrhea, and hypotension [3]. FPIES can be categorized as acute or chronic based on differential symptoms and frequency of exposure to a trigger food [4]. Acute FPIES is defined by repetitive projectile emesis after sporadic ingestion of a trigger food, whereas chronic FPIES exhibits intermittent emesis and failure to thrive due to consistent ingestion [4]. Removal of the suspected trigger food from the diet has been observed to resolve all symptoms within 24 h for acute FPIES and 3–10 days for chronic FPIES [5]. The most common allergen known to cause FPIES is cow’s milk, followed by other prevalent triggers such as soy, eggs, and grains [2, 6]. The first introduction of such allergens has been correlated with the initial presentation of FPIES reactions, where roughly 75% of acute FPIES reactions take place after the first or second consumption of the culprit food [7]. An increase in research and clinical consideration of FPIES has led to the discovery of unique deviations from the standard FPIES triggers and presentations [8]. In this study, we describe a female pediatric patient who developed an acute FPIES allergy to okra after tolerating okra daily for a year.

## Case presentation

A 36-month-old female patient presented initially to the pediatric clinic with a history of delayed, acute projectile emesis and diarrhea, without signs of anaphylaxis following ingestion of gumbo soup beginning at 14-months of age. The patient was born by spontaneous vaginal delivery, exhibiting no complications during gestation or birth. She had no confounding infections, medication, or other food ingestions prior to her first presumed FPIES reaction. The family denied history of any atopic co-morbidities – including any IgE food allergies. Importantly, she previously tolerated gumbo over several months prior to her initial reaction and subsequent ingestion trials produced the same gastrointestinal symptoms consistent with FPIES.

**Fig. 1:**
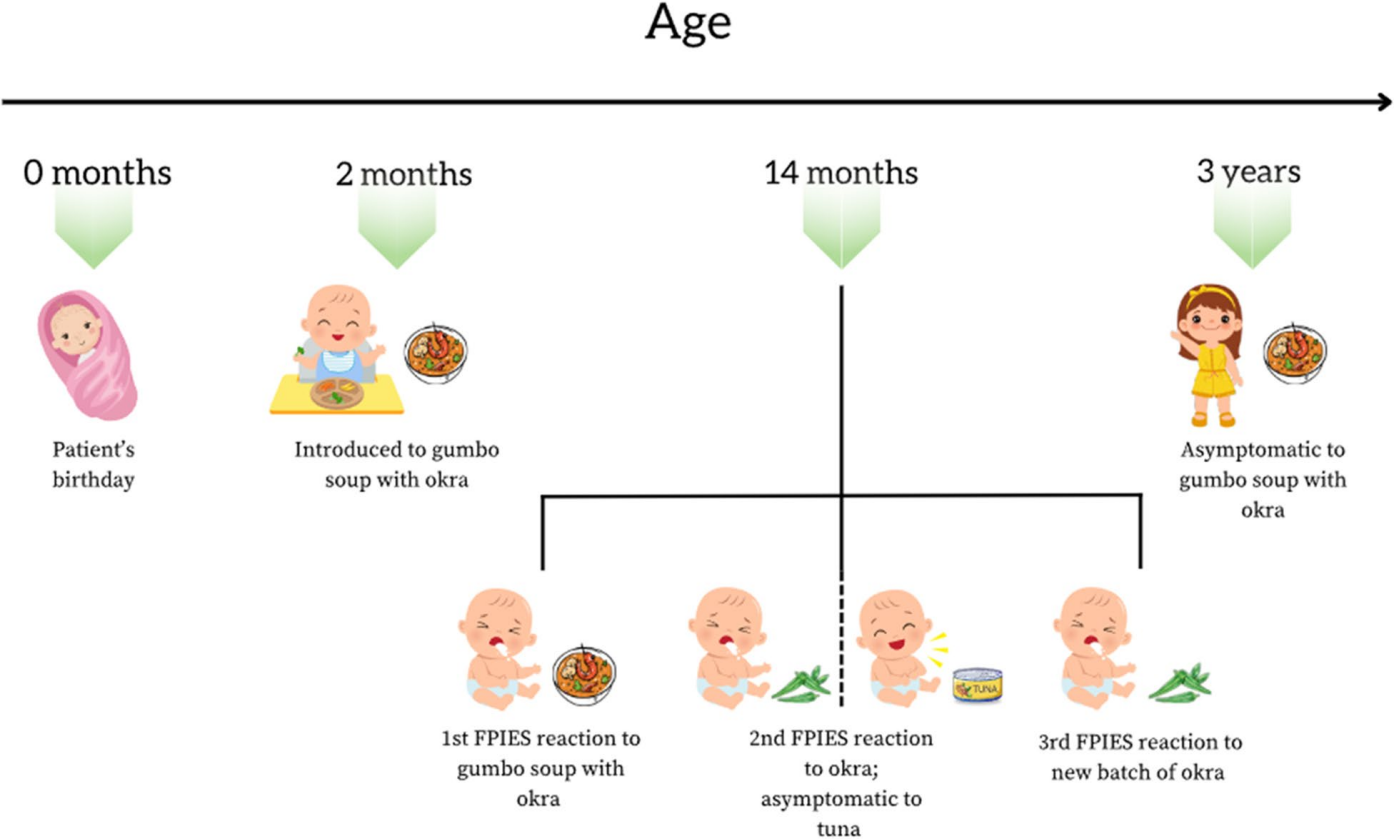
Patient timeline of FPIES to okra

Gumbo, which translates to “okra” in numerous West African dialects, is a thick, okra-based stew containing spices and various seafood ingredients [9]. The patient began consuming small amounts (e.g. ranging from”a taste” to ¼–1/2 cup) of a family recipe of gumbo consisting of okra and tuna at about 2-months of age as was the cultural practice for families from their native West African nation. She consumed her family’s gumbo soup regularly within her diet for a year until she experienced recurrent, projectile emesis and diarrhea 2–3 h after an isolated consumption of ½ a cup of gumbo. She would have over five episodes of projectile emesis, about every 10 min over the course of 2 h, and non-bloody, non-mucoid diarrhea that lasted for about 2 h. No cutaneous or respiratory symptoms were observed to indicate IgE-mediated hypersensitivity. After the resolution of her symptoms, the patient was lethargic, but she responded well to oral hydration with apple juice and tolerated intake of foods high in carbohydrates such as spaghetti and crackers. Emergency room care was not utilized. Within the same month, the family gave the child okra and tuna ingredients from the gumbo soup to consume separately. The patient exhibited no symptoms after consuming the tuna, but did experience recurrent, projectile emesis and diarrhea 1 h after ingesting the okra. The family then bought a fresh batch of okra to determine whether the okra in their possession was problematic, but after consumption, the new okra triggered identical symptoms of emesis and diarrhea for the patient for her 3rd and final reaction. Figure 1 illustrates her clinical course timeline pertinent to the FPIES history.

Based on her history, she was clinically diagnosed with FPIES and advised to avoid okra and all okra-based foods. The family strictly heeded this suggestion and no further incidences of reactions occurred. At the family’s discretion, okra in gumbo soup was slowly reintroduced at 3 years of age, in serving sizes starting at about ¼ cup on a daily basis with good tolerance and the patient has since been asymptomatic since. No other changes in her medical history were noted and she now has no need for any food avoidance measures.

## Discussion

While FPIES reactions can theoretically be caused by any food, cow’s milk is known to be the most prominent trigger [2]. A large 2013 North American study discovered a 67% incidence rate of cow’s milk allergy amongst patients with FPIES [10]. Within the same study, a combination of numerous grains was found to be the second most prevalent at 57%, followed by 41% of FPIES allergies triggered by soy [10]. Fruits also have been known to trigger FPIES, such as bananas, apples, and strawberries, but they are uncommon. Less than 10% of cases reported in the American study have been linked to fruits [6, 10, 11]. While FPIES triggers can vary in frequency depending on the region of study and the participants observed, fruits are typically dwarfed by more dominant triggers such as cow’s milk in the United States and fish in European countries like Greece, Spain, and Italy [11]. To our knowledge, okra has never been formally described as a trigger of FPIES, but as a fruit it should be given clinical consideration as a culprit food if delayed symptoms of vomiting, lethargy, diarrhea,, and in severe cases, hypotension and hypothermia follow its ingestion [3, 8, 12]. The inclusion of okra as a recognized potential FPIES trigger can help reduce the risk of developing chronic FPIES and accelerate the proper diagnosis and appropriate treatment to limit complications associated with dehydration.

While many triggering foods of FPIES have been described, knowledge about the natural history of FPIES remains incomplete. Most FPIES reactions have been found to occur after the first few introductions of the offending food, with studies reporting that 61% to 75% of FPIES reactions occur after the first 2–3 consumptions of the allergen, while others generically report reactions within the first month of introduction to the food trigger [7, 11, 13]. Most current literature discussing FPIES do not focus specifically on the initial presentation of a reaction and lack clarity around the circumstances of the first presentation. In this case, the patient tolerated okra almost daily for a year until she experienced her first FPIES reaction. This notable deviation from the widely-held view of FPIES natural history suggests a need to more broadly consider the disease in patients who once tolerated the food. Due to the variability of FPIES and its prognosis, all foods that elicit characteristic symptoms of the syndrome should be considered as potential triggers attributable to FPIES to reduce potential delays in treatment and management of reactions. It should also be mentioned that different cultures and geographic regions have distinct practices for food introduction [15]. Thus, unique food triggers may reflect regional practices of early food introduction. Overall, FPIES has a good outcome with many studies reporting a 60% rate of resolution for any food [16].

Treatment of FPIES involves removing the known allergen from the diet to avoid subsequent reactions [8]. If ingestion of the food were to occur and evoke a mild reaction of 1–2 episodes of vomiting, parents should offer oral fluids for rehydration [6]. For severe reactions with more than three episodes, lethargy, and features of moderate to severe dehydration, patients should seek medical intervention including intravenous fluids for rehydration [6, 13]. Serotonin release in the gastrointestinal tract in response to the activation of the purinergic pathway offers a possible explanation for the vomiting observed in patients with FPIES. Furthermore, Ondansetron, a serotonin receptor antagonist, has proven effective in the treatment of FPIES-induced vomiting. [17] Oral ondansetron can be offered for mild cases managed at home, with a repeat dose offered if vomiting occurs within ten minutes of the initial dose. IM and IV ondansetron are reserved for severe cases managed in the hospital setting. [6, 13]. Oral food challenges according to published guidelines, may be attempted 12–18 months after the last reaction to determine whether tolerance to the trigger food has developed and FPIES has resolved [13, 14]. Patient education and support are critical to early recognition and prompt management of FPIES to prevent the development of complications related to dehydration in affected patients.

## Conclusion

Recently, awareness about the varied nature of FPIES clinical presentation has come to light. This case is the first to describe FPIES to the fruit okra in a child that developed over a 12-month span after previously tolerating the food. This case serves to emphasize the importance of understanding the range of FPIES symptoms to improve recognition and expedite management.

## Data Availability

Not applicable.

## Abbreviations

FPIES: Food protein-induced enterocolitis syndrome
non-IgE: Non-immunoglobulin E
